# Microbial Community Changes in the Rhizosphere Soil of Healthy and Rusty *Panax ginseng* and Discovery of Pivotal Fungal Genera Associated with Rusty Roots

**DOI:** 10.1155/2020/8018525

**Published:** 2020-01-15

**Authors:** Xuemin Wei, Xiaoyue Wang, Pei Cao, Zitong Gao, Amanda Juan Chen, Jianping Han

**Affiliations:** Institute of Medicinal Plant Development, Chinese Academy of Medical Sciences & Peking Union Medical College, Beijing 100193, China

## Abstract

*Panax ginseng* Meyer, a valuable medicinal plant, is severely threatened by rusty root, a condition that greatly affects its yield and quality. Studies investigating the relationship between soil microbial community composition and rusty roots are vital for the production of high-quality ginseng. Here, high-throughput sequencing was employed to systematically characterize changes in the soil microbial community associated with rusty roots. Fungal diversity was lower in the soils of rusty root-affected *P. ginseng* than in those of healthy plants. Importantly, principal coordinate analysis separated the fungal communities in the rhizosphere soils of rusty root-affected ginseng from those of healthy plants. The dominant bacterial and fungal genera differed significantly between rhizosphere soils of healthy and rusty root-affected *P. ginseng*, and linear discriminant analysis effect size (LEfSe) further indicated a strong imbalance in the soil microbial community of diseased plants. Significantly enriched bacterial genera (including *Rhodomicrobium*, *Knoellia*, *Nakamurella*, *Asticcacaulis*, and *Actinomadura*) were mainly detected in the soil of rusty root-affected *P. ginseng*, whereas significantly enriched fungal genera (including *Xenopolyscytalum*, *Arthrobotrys*, *Chalara*, *Cryptococcus*, and *Scutellinia*) were primarily detected in the soil of healthy plants. Importantly, five fungal genera (*Cylindrocarpon*, *Acrophialophora*, *Alternaria*, *Doratomyces*, and *Fusarium*) were significantly enriched in the soil of rusty root-affected plants compared with that of healthy plants, suggesting that an increase in the relative abundance of these pathogenic fungi (*Cylindrocarpon*, *Alternaria*, and *Fusarium*) may be associated with ginseng rusty roots. Additionally, this study is the first to report that an increase in the relative abundances of *Acrophialophora* and *Doratomyces* in the rhizosphere of *P. ginseng* may be associated with the onset of rusty root symptoms in this plant. Our findings provide potentially useful information for developing biological control strategies against rusty root, as well as scope for future screening of fungal pathogens in rusty roots of *P. ginseng*.

## 1. Introduction

*Panax ginseng* Meyer, belonging to the Araliaceae family, is one of the most widely known medicinal plants. *P. ginseng* is currently consumed in 35 countries globally, primarily in East Asia, and especially in Korea and China [1]. Ginseng, which commonly refers to the dry roots and rhizomes of *P. ginseng*, has been used as a valuable and important folk medicine in China for more than 2000 years [2]. Furthermore, the global popularity of ginseng has increased greatly over the past few decades owing to its potent pharmacological activities, including its antiaging, antidiabetic, immunoregulatory, anticancer, and neuroregulatory properties [3–7]. The current global market for ginseng is estimated to be approximately $2,084 million [8]. *P. ginseng* is a herbaceous perennial plant, and a cultivation period of 4 to 6 years is required to obtain high-quality ginseng with good medicinal properties. However, after 4 years of consecutive cultivation, there is an increase in soilborne diseases that leads to dramatic yield losses and a decline in the quality of ginseng [9–11]. Rusty root, one of the most destructive root rot diseases affecting *P. ginseng*, is characterized by small or large reddish-brown spots on the surface of roots. Because rusty roots result in a significant annual decline in yield of between 20% and 30% [12] and cause substantial economic losses, this rusty root disease has attracted considerable attention [13, 14].

Soil microorganisms play vital roles in the maintenance of soil function [15] and significantly influence agricultural soil productivity, plant growth, and crop quality [16–18]. Numerous studies have suggested that microbial diversity can serve as an indicator of disease susceptibility in medicinal plants, and microbial communities have important roles in the maintenance of plant health and soil fertility [19–21]. For example, a lack of diversity among beneficial microbial communities like that of *Alteromonadales*, *Burkholderiales*, and *Flavobacteriales*, may be an important factor contributing to the decline in peanut yield under continuous cropping [22]. Additionally, plant death rate and fungal diversity are significantly negatively correlated, and the relative abundances of *Fusarium oxysporum* and *Phaeosphaeria rousseliana* are positively associated with the death rate of *Panax notoginseng* (*P* < 0.05) [23]. Li et al. found that plant disease suppressing or growth-promoting bacteria such as *Pseudomonas*, *Burkholderia*, and *Bacillus*, tended to be rare in *P. ginseng* rhizosphere soils with an increase in years of monoculture [24]. Consequently, microbial community diversity in combination with community composition, especially of key functional species, may be a meaningful indicator of soil health [19].

The relationship between rhizospheric microorganisms and rusty root symptoms in *P. ginseng* has been extensively investigated. Several studies have demonstrated that rusty root can be caused by bacteria, fungi, or a combination of both [25, 26]. For example, Farh et al. reported that the rusty root of *P. ginseng* was caused by *Cylindrocarpon destructans* var. *destructans* [11], while Lu et al. investigating the pathogenicity of *Ilyonectria robusta* against the root of *P. ginseng* using repeated inoculation, highlighted that *I. robusta* could be the causative agent of rusty root of *P. ginseng* in China [27]. Recent studies have also indicated that the *Ilyonectria* fungus was the most likely cause of rusty root in *P. ginseng*, as this pathogen was found to be enriched in the rhizosphere soils of plants exhibiting rusty root symptoms [11, 25]. Furthermore, Guan et al. isolated *Fusarium redolens* from diseased roots of *P. ginseng* and reported this pathogenic fungus as a new causative agent of *P. ginseng* root rot in China [28]. However, in 2006, *Rhexocercosporidium panacis* was reported to infect the roots of *P. quinquefolius* and cause rusty root [29], while in 2014 it was first reported to also cause the rusty roots of *P. ginseng* [30]. Even though these and other studies have revealed the relationship between several pathogenic species and rusty root, the causative agent(s) of this rusty root in ginseng remains unclear. Additionally, relatively few studies have evaluated the relationship between rusty root and changes in the microbial community of the rhizosphere of *P. ginseng*. Most investigations on rusty root have only considered one microbial species, while not all predominant microbial groups can be detected due to the limitations of traditional molecular methods, such as polymorphic DNA amplification [31] and denaturing gradient gel electrophoresis [32], and the results obtained using these methods have been inconsistent. Therefore, whether rusty root is caused by multiple pathogenic microorganisms, rather than just one, remains unknown. Because a wide diversity of microorganisms, including beneficial, harmful, and neutral microbes, are present in rhizosphere soils, they are likely to simultaneously interact with plant roots in the rhizosphere [33]. Indeed, several pathogens are known to act in concert to induce disease in plants. Therefore, it is important to elucidate the relationships between rusty roots and microorganisms, including investigating changes in the composition of the entire microbiome coexisting in the rhizosphere of *P. ginseng.* Additionally, recent advances in metagenomic-based approaches [34–36] have expanded our ability to investigate the differences in the composition of rhizospheric microbial communities between healthy and rusty root-affected *P. ginseng*.

In this study, 16S and internal transcribed spacer (ITS) ribosomal RNA (rRNA) sequencing was used to detect and compare changes in the diversity and composition of bacterial and fungal communities in rhizosphere soils of both healthy and rusty root-affected ginseng. The results will be useful for understanding the contribution of soil microecology to rusty root etiology, as well as for promoting the development of efficient biological control strategies and the sustainability of the traditional medicine industry.

## 2. Materials and Methods

### 2.1. Sample Processing

All soil samples assayed in this study were collected from Fusong County, Jilin Province, China (42°23′N, 127°05′E), in October 2017. This region has a temperate continental monsoon climate with an annual precipitation of approximately 800 mm. Fusong County is the best known ginseng-producing region in China and is known as the hometown of *P. ginseng*. Soil samples were collected from the root zones of healthy plants (denoted as H) and those of rusty root-affected plants (denoted as D) located in a 4-year-old ginseng plantation. These plantations used a ridging cultivation pattern, and the preceding crop was also *P. ginseng*. The experiment was conducted with three replicates, and the area of each replicated plot was 1.5 m × 10 m. Each sample consisted of a mixture of five healthy or five rusty root-affected *P. ginseng* plants from the same plot. Ginseng plants with dark-green leaves, normal stems, and no reddish-brown areas on the root surface were considered healthy, while those with a wilted stem, non-dark-green-colored leaves, hollow roots, and reddish-brown spots on the root surface were considered diseased. *P. ginseng* seedlings were removed from the plots, and the roots were gently shaken to remove loosely adhering soil. The soil adhering tightly to the surface of both H and D ginseng roots was brushed off and pooled in sterile plastic bags. All soil samples were homogenized by passing through a 2 mm sieve and stored at −80°C until further processing. The physical and chemical properties of the soils were as follows: pH, 5.11; available nitrogen, 310.89 mg·kg^−1^; available phosphorus, 117.54 mg·kg^−1^; and available potassium, 359.42 mg·kg^−1^.

### 2.2. DNA Extraction and PCR Amplification

Total soil DNA was extracted from 0.1 g of soil sample using a Mo Bio Powersoil DNA Kit (Mo Bio Laboratories, Carlsbad, CA, USA) according to the manufacturer's protocol. The successful extraction and purity of the genomic DNA were verified by 0.8% agarose gel electrophoresis with 1 × TAE buffer. The extracted DNA was stored at −20°C until use. For each sample, the bacterial 16S rRNA gene was amplified using the bacterial-specific primer pair 515F (5′-GTGCCAGCMGCCGCGG-3′)/907R (5′-CCGTCAATTCMTTTRAGTTT-3′) (V4 + V5 regions) [37, 38]; and the ITS region of the fungal rRNA gene was amplified using the fungal-specific primer pair ITS1F (5′-CTTGGTCATTTAGAGGAAGTAA-3′)/ITS2R (5′-GCTGCGTTCTTCATCGATGC-3′) [39]. Polymerase chain reaction (PCR) amplification and purification were performed as previously described [40]. The purified PCR products were quantified using a QuantiFluor™-ST system (Promega, USA), and the amplicons were pooled in equimolar ratios for sequencing.

### 2.3. High-Throughput Sequencing and Statistical Analyses

Pooled DNA products were used to construct an Illumina paired-end library and subsequently paired-end sequenced (2 × 250) on an Illumina HiSeq 2500 platform (Shanghai Biozeron Co., Ltd, Shanghai, China) according to the manufacturer's protocol. Data were demultiplexed and quality filtered using QIIME (QIIME 1.9, http://qiime.org/scripts/assign_taxonomy.html) according to the standard pipeline [41]; after trimming, FASTQ files were transformed to FASTA format. Briefly, sequences with ≥97% identity were assigned to the same operational taxonomic unit (OTU) using UPARSE [42]. Chimeric sequences were identified and removed by the *de novo* method using UCHIME [43]. The taxonomic identities of the bacteria and fungi were determined using the Silva (http://www.arb-silva.de) [44] and Unite 130 databases (http://unite.ut.ee/index.php) [45], respectively. Alpha diversity analysis of the bacterial and fungal communities was performed to determine Shannon (*H′*), Chao I, and abundance-based coverage estimator (Ace) diversity indices, using a modified version of a previously described procedure [46]. Principal coordinate analysis (PCoA) was used to compare groups of samples based on unweighted UniFrac distance metrics. Linear discriminant analysis (LDA) effect size (LEfSe) [47, 48] was applied to further identify bacterial and fungal genera among all OTUs with statistically different abundances between different groups. Differences were considered significant with an LDA score >2 or 3 and *P* < 0.05. The raw reads generated in the study have been submitted to the NCBI's SRA. Accession: PRJNA589989.

### 2.4. Statistical Analyses

IBM SPSS Statistics 21 software was employed to compare microbial diversity indices and the relative abundance of soil microbial communities. Variables for all treatment replicates were considered and subjected to ANOVA. The data are presented as the mean ± SE (*n* = 3). Mean values were considered significant at *P* < 0.05 as assessed by *t*-tests.

## 3. Results

### 3.1. Amplicon Sequencing and Community Diversity Overview

**Figure 1:**
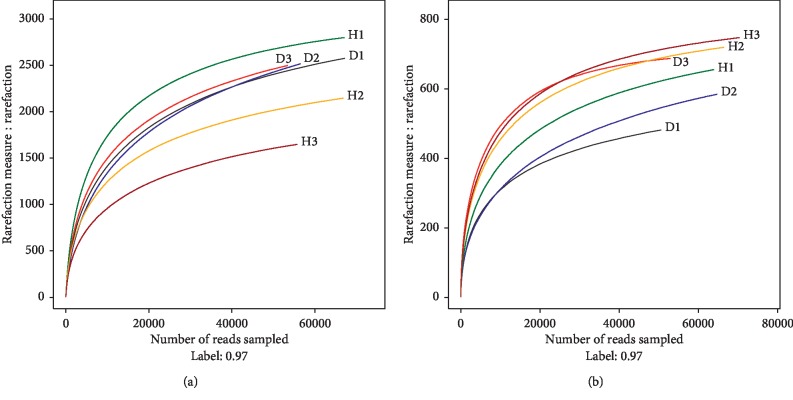
Bacterial (a) and fungal (b) rarefaction curves for all samples at a 97% OTU sequence similarity threshold.

**Figure 2:**
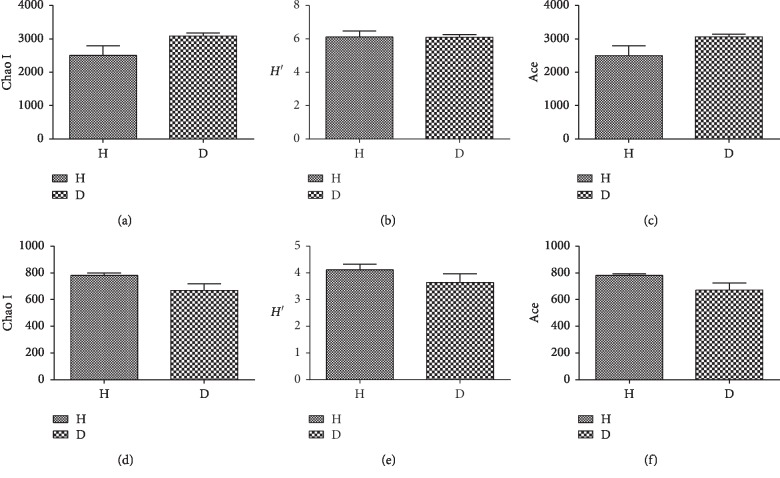
Bacterial and fungal diversity in the rhizosphere of healthy and rusty root-affected *Panax ginseng*. (a), (b), and (c) show the Chao I *H′*, and Ace index values, respectively, for the bacterial community. (d), (e), and (f) show the Chao I *H′*, and Ace index values, respectively, for the fungal community. All values are presented as means ± SE (*n* = 3). H: healthy plants; D: diseased plants.

A total of 386,082 classifiable bacterial sequences and 377,887 classifiable fungal sequences were obtained from six soils after quality control filtering, with a mean number of 64,347 and 62,981 classifiable sequences per sample, respectively. In contrast, 4,323 bacterial and 2,254 fungal OTUs were identified among all soil samples at a 97% sequence similarity cut off. Rarefaction curves were used to evaluate OTU saturation and indicated that the sequencing efforts were sufficient for this study, as the number of OTUs was close to saturation (Figure 1). The alpha diversity of the bacterial and fungal microbiomes of each sample was estimated using Chao I, *H′*, and Ace indices (Figure 2). Chao I and Ace indices were used to estimate the richness of all the soil samples, while *H′* was used to estimate the diversity of all the soil samples. For bacteria, the mean values for the Chao I and Ace indices were higher in D soils (three samples) (Chao I, 3084.68; Ace, 3062.58) than in H soils (three samples) (Chao I, 2504.04; Ace, 2496.58), whereas the mean *H′* values were lower in D soils (6.09) than in H soils (6.11). For fungi, all the index values were lower in D soils (Chao I, 669.02; Ace, 671.43; *H′*, 3.24) than in H soils (Chao I, 780.63; Ace, 780.24; *H′*, 4.11). Compared with H soils, the values for fungal Chao I, Ace, and *H′* indices in D soils were decreased by 14.30%, 13.95%, and 21.19%, respectively.

### 3.2. Changes in Bacterial Community Structure in the Rhizosphere Soil of Rusty Root-Affected Ginseng

**Figure 3:**
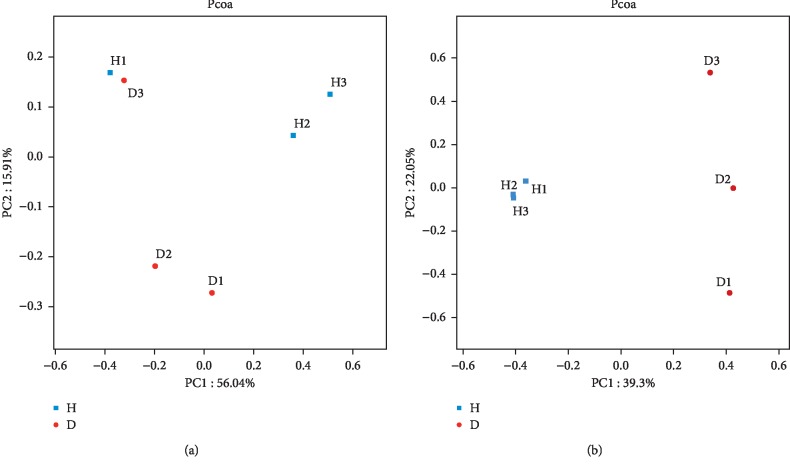
Principal coordinate analyses (PCoA) for bacteria (a) and fungi (b) in the rhizosphere soil of healthy and rusty root-affected *Panax ginseng.* All values are presented as means ± SE (*n* = 3). H: healthy plants; D: diseased plants.

**Figure 4:**
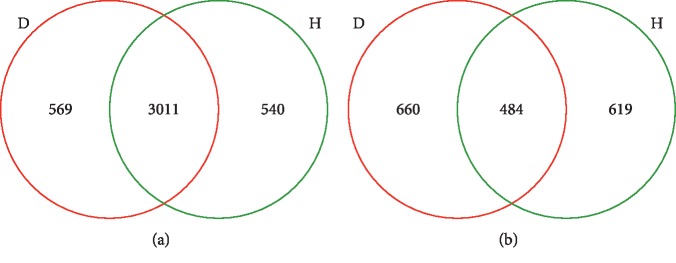
Venn diagram for bacterial (a) and fungal (b) communities. Venn diagram showing the number of shared and unique operational taxonomic units (≥97% similarity) among the rhizosphere soils of healthy and rusty root-affected *Panax ginseng*. H: healthy plants; D: diseased plants.

PCoA ordination revealed differences in the bacterial communities between the H and D soils. The second principal component (15.91% contribution) showed that the bacterial communities in the D soil samples (except for D3) were different from those of the H soils (Figure 3(a)). A Venn diagram indicated that 3,011 bacterial OTUs were shared between H and D soils, and that 540 and 569 OTUs were found exclusively in H and D soils, respectively (Figure 4(a)).

**Figure 5:**
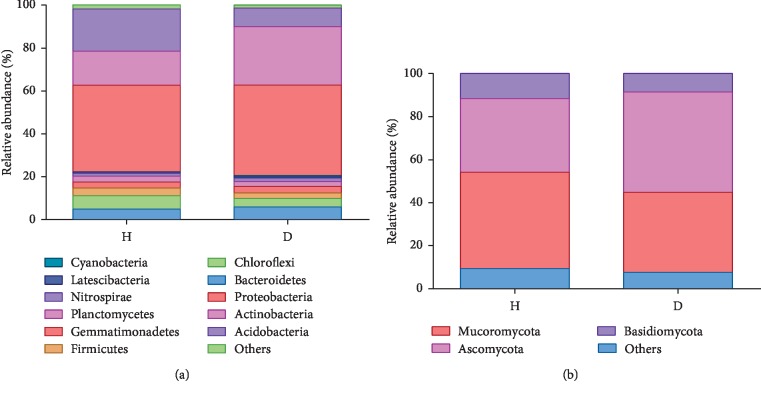
Taxonomic classification at the phylum level of bacterial (a) and fungal (b) reads retrieved from healthy and rusty root-affected *Panax ginseng*. The bar marked “Others” represents the relative abundance of all phyla not specifically listed. All values are presented as means ± SE (*n* = 3). H: healthy plants; D: diseased plants.

The predominant phyla in all the soil samples (H and D) were *Proteobacteria*, *Actinobacteria*, *Acidobacteria*, *Bacteroidetes*, *Chloroflexi*, *Firmicutes*, *Gemmatimonadetes*, *Planctomycetes*, *Nitrospirae*, and *Latescibacteria*, with average abundances of 41.25%, 21.53%, 14.22%, 5.49%, 5.05%, 3.09%, 2.91%, 2.52%, 1.49%, 0.53%, and 0.36%, respectively (Figure 5(a)). Among the 11 phyla, the average relative abundances of *Proteobacteria*, *Actinobacteria*, *Bacteroidetes*, *Gemmatimonadetes*, *Nitrospirae*, and *Cyanobacteria* were increased to varying degrees in the D soils compared with those in H soils, but the opposite was observed for *Acidobacteria*, *Chloroflexi*, *Firmicutes*, *Planctomycetes*, and *Latescibacteria*. The 9 most abundant bacterial phyla (with a relative abundance of more than 1.00%) in H soil samples were *Proteobacteria*, *Acidobacteria*, *Actinobacteria*, *Chloroflexi*, *Bacteroidetes*, *Planctomycetes*, *Firmicutes*, *Gemmatimonadetes*, and *Nitrospirae*, with average relative abundances of 40.34%, 19.69%, 15.87%, 6.20%, 4.99%, 2.80%, 3.58%, 2.79%, and 1.28%, respectively. However, the top nine most abundant (with a relative abundance of more than 1.00%) bacterial phyla assemblages in D soil samples were *Proteobacteria*, *Actinobacteria*, *Bacteroidetes*, *Acidobacteria*, *Firmicutes*, *Chloroflexi*, *Planctomycetes*, *Gemmatimonadetes*, and *Nitrospirae*, which accounted for 42.15%, 27.20%, 5.99%, 8.75%, 2.60%, 3.91%, 2.23%, 3.03%, and 1.70%, respectively, of the total population in all the D soils.

**Figure 6:**
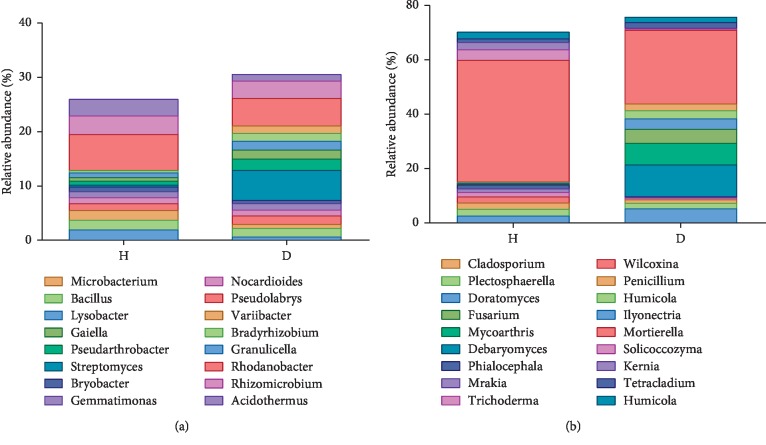
Taxonomic classification at the genus level of bacterial (a) and fungal (b) reads retrieved from healthy and rusty root-affected *Panax ginseng*. H: healthy plants; D: diseased plants.

At the genus level, the 10 most abundant bacteria in H soil samples were *Rhodanobacter*, *Rhizomicrobium*, *Acidothermus*, *Granulicella*, *Bradyrhizobium*, *Variibacter*, *Pseudolabrys*, *Nocardioides*, *Gemmatimonas*, and *Bryobacter*, with average relative abundances of 6.63%, 3.42%, 3.04%, 1.91%, 1.78%, 1.78%, 1.26%, 1.10%, 1.10%, and 0.89%, respectively. The 10 most abundant bacterial genera in D soil samples were *Streptomyces*, *Rhodanobacter*, *Rhizomicrobium*, *Pseudarthrobacter*, *Gaiella*, *Lysobacter*, *Bradyrhizobium*, *Pseudolabrys*, *Bacillus*, and *Microbacterium* (5.51%, 5.09%, 3.17%, 2.08%, 1.66%, 1.63%, 1.59%, 1.57%, 1.43%, and 1.38%, respectively) (Figure 6(a)).

### 3.3. Changes in Fungal Community Structure in the Rhizosphere Soil of Rusty Root-Affected Ginseng

PCoA analysis revealed that fungal communities from different soil samples clustered according to their groups (Figure 3(b)). The first principal component (39.30% contribution) differentiated the fungal communities of D soils from those of H soils. The fungal communities in the H soils were similar to each other, but markedly different from those in the D soils. Venn diagram analysis revealed that H and D soils shared 484 fungal OTUs, whereas 619 and 660 OTUs were exclusive to H and D soils, respectively (Figure 4(b)). The predominant phyla across all H and D soil samples were *Mucoromycota*, *Ascomycota*, and *Basidiomycota*, with mean abundances of 35.98%, 48.71%, and 8.00%, respectively (Figure 5(b)). The 3 phyla with the highest relative abundances in the H soil samples were *Mucoromycota*, *Ascomycota*, and *Basidiomycota*, with average abundances of 44.79%, 34.28%, and 11.54%, respectively, while the 3 most abundant fungal phyla in the D soil samples were *Ascomycota, Mucoromycota, and Basidiomycota,* accounting for 63.14%, 27.17%, and 4.46% of the total population in all the D soils, respectively.

At the genus level, the 10 most abundant fungi in the H soil samples were *Mortierella*, *Solicoccozyma*, *Kernia*, *Ilyonectria*, *Humicola*, *Penicillium*, *Wilcoxina*, *Trichoderma*, *Mrakia*, and *Phialocephala*, with average relative abundances of 44.71%, 3.89%, 2.66%, 2.51%, 2.46%, 2.33%, 2.27%, 1.56%, 1.40%, and 1.37%, respectively. However, the 10 most abundant fungal genera in the D soil samples were *Mortierella*, *Debaryomyces*, *Mycoarthris*, *Ilyonectria*, *Fusarium*, *Doratomyces*, *Plectosphaerella*, *Cladosporium*, *Tetracladium*, and *Humicola*, with average relative abundances of 27.14%, 11.72%, 7.95%, 5.20%, 5.15%, 3.84%, 2.97%, 2.48%, 2.03%, and 1.98%, respectively (Figure 6(b)).

### 3.4. Strong Imbalance in the Bacterial Community (at the Genus Level) of Rusty Root-Affected Ginseng

**Figure 7:**
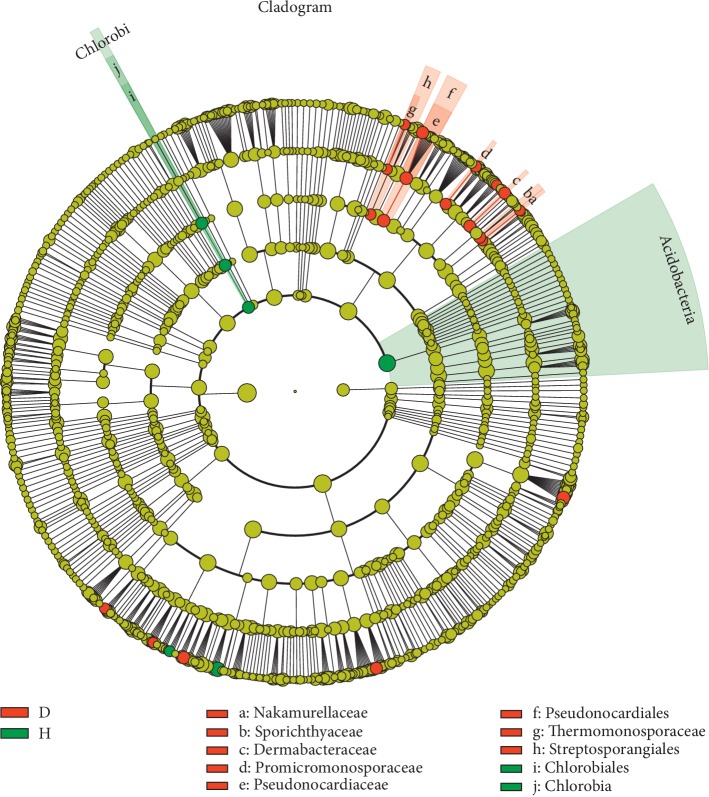
Linear discriminant analysis effect size (LEfSe) for bacterial taxa between soils of healthy and rusty root-affected *Panax ginseng*. Cladogram showing significantly enriched bacterial taxa (from phylum to family level). Significant differences are defined at *P* < 0.05 and an LDA score >2.0. H: healthy plants; D: diseased plants.

The LEfSe algorithm was applied to identify bacterial taxa from all the OTUs that showed significantly different abundances between the H and D soil samples (Table 1, Figure 7). In total, 2 orders (*Pseudonocardialese* and *Streptosporangiales*), 6 families (*Pseudonocardiaceae*, *Dermabacteraceae*, *Sporichthyaceae*, *Thermomonosporaceae*, *Nakamurellaceae*, and *Promicromonosporaceae*), and 11 genera were more abundant in D soils than in H soils. Specifically, at the genus level, *Rhodomicrobium*, *Knoellia*, *Nakamurella*, *Asticcacaulis*, *Actinomadura*, *Collimonas*, *Pseudonocardia*, and *Dokdonella* were enriched in D soils, with relative rates of increases in abundance of 64.71%, 246.67%, 190.48%, 173.91%, 642.86%, 215.73%, 185.09%, and 151.09%, respectively (*P* < 0.05). In addition, *Promicromonospora*, *Brachybacterium*, and *Phyllobacterium* were more abundant in D soil samples than in H soil samples (*P* < 0.05). However, the relative abundances of 2 phyla (*Acidobacteria* and *Chlorobi*), 1 class (*Chlorobia*), 1 order (*Chlorobiales*), and 2 genera were lower in the D soils than in the H soils. Compared with H soils, the 2 least abundant genera in D soil samples were *Hartmannibacter* and *Variibacter*, with relative rates of decreases in abundance of 971.43% and 104.79%, respectively (*P* < 0.05).

### 3.5. Fungal Genera Associated with *P. ginseng* Rusty Roots

**Figure 8:**
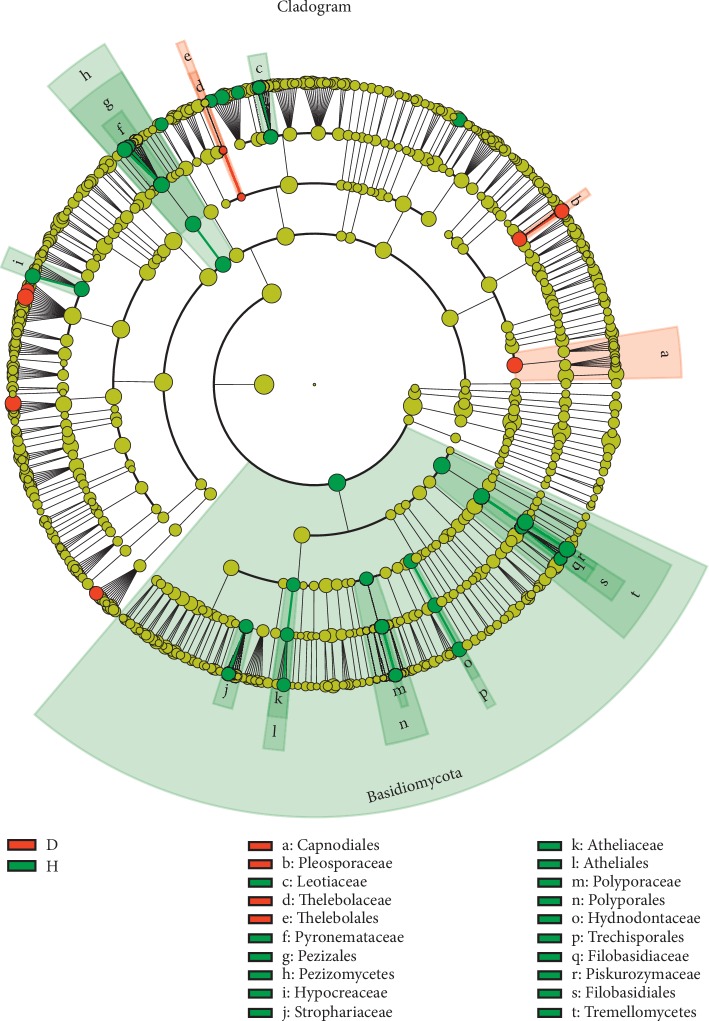
Linear discriminant analysis effect size (LEfSe) for fungal taxa between soils of healthy and rusty root-affected *Panax ginseng*. Cladogram showing significantly enriched fungal taxa (from phylum to family level). Significant differences are defined at *P* < 0.05 and an LDA score >3.0. H: healthy plants; D: diseased plants.

For fungi, LEfSe analysis also indicated significant differences in relative taxonomic abundances between H and D soil samples (Table 2, Figure 8). In total, the relative abundances of 1 order (*Capnodiales*), 1 family (*Pleosporaceae*), and 5 genera were enriched in D soils when compared with H soils. Importantly, the relative abundances of 5 genera (*Cylindrocarpon*, *Acrophialophora*, *Alternaria*, *Doratomyces*, and *Fusarium*) increased from 0.04% to 0.26%, 0.01% to 0.41%, 0.09% to 0.83%, 0.38% to 3.84%, and 0.33% to 5.15%, respectively (*P* < 0.05) compared with those in H soils. In addition, 1 phylum (*Basidiomycota*), 2 classes (*Tremellomycetes* and *Pezizomycetes*), 5 orders (*Filobasidiales*, *Pezizales*, *Trechisporales*, *Atheliales*, and *Polyporales*), 9 families (*Polyporaceae*, *Atheliaceae*, *Piskurozymaceae*, *Piskurozymaceae*, *Hydnodontaceae*, *Hypocreaceae*, *Strophariaceae*, *Leotiaceae*, and *Filobasidiaceae*), and 15 genera were less abundant in D soils than in H soils. In total, 15 genera were less abundant in D soils than in H soils, namely, *Xenopolyscytalum*, *Arthrobotrys*, *Chalara*, *Cryptococcus*, *Scutellinia*, *Neobulgaria*, *Galerina*, *Piloderma*, *Ganoderma*, *Hamigera*, *Trichoderma*, *Phialocephala*, *Trechispora*, *Wilcoxina*, and *Solicoccozyma* (*P* < 0.05).

## 4. Discussion

In this study, to obtain a preliminary understanding of the relationship between the microbial community composition and rusty root-affected ginseng, we systematically compared the composition of the bacterial and fungal communities between rhizosphere soils of healthy and diseased ginseng using Illumina MiSeq high-throughput sequencing. Overall, Chao I, *H′*, and ACE index values for bacteria and fungi (except bacterial Chao I and Ace) were lower in rhizosphere soils of rusty root-affected ginseng than in those of healthy plants. Our results for fungal diversity were in agreement with those of previous studies that have indicated that microbial diversity was greater in the soil of healthy plants than in that of diseased plants [19, 49]; however, this was not the case for bacterial diversity. The results obtained in the present study are instead consistent with those of Dong et al. [23], who reported that fungal diversity could serve as a bioindicator of soil health status, whereas bacterial diversity showed an increasing trend under continuous cropping of *P. notoginseng*. In addition to plant health, the diversity of rhizosphere microbial communities is also shaped by soil type, plant species, pedoclimate, climate, and season, as well as several other biotic and abiotic factors [50–52]. All these factors might explain why, in the present study, bacterial diversity was lower in the rhizosphere of healthy *P. ginseng* plants than in that of diseased plants. PCoA analysis suggested that rusty root symptoms were likely to induce changes in soil microbial community structure, especially in that of the fungal community. While the PCoA analysis for bacteria showed that the community composition in sample D3 was more like that of H samples than that of the other D samples, Wu et al. also revealed that the community structure in the rhizosphere soil of several healthy *P. notoginseng* samples was more similar to that of diseased plants than to that of other healthy plants [19]. More research is needed to reveal the possible reasons for the differences in microbial community structure in rhizosphere soils of diseased plants.

Further analysis revealed a strong imbalance in the composition of bacterial and fungal microbial communities between the rhizosphere soils of healthy and rusty root-affected ginseng. Indeed, the bacterial and fungal soil microbiomes exhibited a severe imbalance between healthy and diseased plants, which was mainly attributed to differences in the dominant genera and their relative abundances. The dominant bacterial and fungal genera in the soil of healthy ginseng plants were considerably different from those of rusty root-affected plants. Moreover, LEfSe showed that 11 bacterial genera were more abundant in the rhizosphere soil of rusty root-affected ginseng than in that of healthy ginseng, while the opposite was true for only 2 bacterial genera (*P* < 0.05, LDA >2.00). Interestingly, 5 fungal genera were enriched in the rhizosphere soil of rusty root-affected ginseng compared to that of healthy ginseng, whereas the opposite was true for 15 fungal genera (*P* < 0.05, LDA >3.00). Soil microbial diversity and the composition of microbial communities play important roles in maintaining soil ecosystem function, health, and quality [13, 27], and disease occurrence in medicinal plants is governed largely by imbalances in soil microbial diversity and community composition [53, 54]. Because rusty root has severely threatened the sustainable development of the *P. ginseng* industry, numerous studies have investigated the relationship between soil microbes and rusty roots; however, most studies have considered only one or two pathogenic fungi that are involved in the occurrence of rusty root in *P. ginseng*, such as *Alternaria panax* Whetz. [55] and *Cylindrocarpon destructans* [56]. In addition, Li et al. [57] showed that relative abundances of putative pathogens, such as *Fusarium*, *Gibberella*, and *Nectriaceae_unclassified*, were higher in fields treated with phenolic acids than in those of *P. ginseng* consecutive monoculture. However, the study by Li et al. was not a systematic analysis of the relationship between rusty root and changes in microbial communities, even though they did investigate changes in fungal community composition using Illumina MiSeq sequencing. Our study filled this gap in systematic research on changes in the microbial community composition of the soil of rusty root-affected *P. ginseng*.

Most soilborne pathogenic microorganisms that cause plant root diseases are fungi, and they pose a serious threat to the soil microecological balance, plant health, and crop-based income [58–61]. In our study, LEfSe indicated that five fungal genera, namely, *Cylindrocarpon*, *Acrophialophora*, *Alternaria*, *Doratomyces*, and *Fusarium*, were considerably more abundant in the rhizosphere soils of rusty root-affected ginseng than in those of healthy ginseng (*P* < 0.05). Interestingly, three of these five genera (*Cylindrocarpon*, *Alternaria*, and *Fusarium*) have been widely reported to be closely related to the occurrence of root diseases in medicinal plants of the genus *Panax*, such as *P. ginseng*, *P. notoginseng*, and *P. quinquefolius.* Root rot diseases of *P. notoginseng* and *P. quinquefolius* are due primarily to two species of *Fusarium*, *F. solani,* and *F. oxysporum* [62, 63]; moreover, a soilborne pathogenic fungus of the genus *Cylindrocarpon*, *C. destructans*, can cause primary root rot or rusty root symptoms in ginseng (*P. ginseng* and *P. notoginseng*) [55, 64]. A pathogenic species of the genus *Alternaria*, *A. panax* Whetz, causes Alternaria panax disease, one of the most commonly occurring and harmful diseases in ginseng (*P. ginseng* and *P. quinquefolius*) [13, 56]. In contrast, to the best of our knowledge, there are no reports of *Acrophialophora* and *Doratomyces* species causing rusty root in *P. ginseng.* However, *D. stemonitis* is responsible for the brown rot of *Solanum tuberosum*, and the symptoms (dark-brown lesions that appear on the surface of diseased *S. tuberosum* tubers followed by tuber rot) are similar to those of ginseng root rot [65]. To date, there are no reports of species of the genus *Acrophialophora* being associated with plant diseases. Soil microbial communities contain numerous pathogenic, nonpathogenic, and symbiotic microorganisms that simultaneously interact with plant roots. Similarly, several soilborne pathogens act in concert to induce disease in plants [66], as supported by our results. Consequently, we concluded that the significant increase in the relative abundance of several pathogenic fungi, such as *Cylindrocarpon*, *Alternaria*, and *Fusarium*, may act together to induce rusty root in *P. ginseng*. LEfSe of high-throughput sequencing data revealed the fungal genera enriched in the rhizosphere soil of rusty root-affected ginseng, many of which were pathogenic species that cause disease in ginseng (*P. ginseng*, *P. notoginseng*, and *P. quinquefolius*). This indicates that LEfSe may be a useful tool for identifying key fungal taxa related to root diseases of *P. ginseng* from high-throughput sequencing data. In view of these observations, this study is the first to indicate that an increase in the relative abundance of *Acrophialophora* and *Doratomyces* in the rhizosphere soil of *P. ginseng* may be associated with the onset of rusty roots in this plant. More research is required to verify this hypothesis and identify the strains related to rusty root of ginseng. Our study also provides potentially useful information for developing biological control strategies against rusty root to mitigate significant economic losses in the ginseng industry.

## 5. Conclusions

In summary, high-throughput sequencing revealed the dynamics of the microbial community associated with *P. ginseng* rusty roots. The present study indicated that bacterial and fungal community structures in the rhizosphere soils of rusty root-affected *P. ginseng* are significantly different from those of healthy plants. Fungal diversity was found to be higher in the soils of healthy *P. ginseng* than in those of rusty root-affected plants. The relative abundances of several pathogenic fungi, such as *Cylindrocarpon*, *Fusarium*, and *Alternaria*, were significantly higher in the rhizosphere soils of rusty root-affected plants than in those of healthy plants. In addition, this study is the first to highlight that a significant increase in the relative abundances of *Acrophialophora* and *Doratomyces* may be associated with the onset of rusty root in *P. ginseng*. Our work will be of great significance for biological control of rusty roots, as well as for the management of *P. ginseng* cultivation.

## Figures and Tables

**Table 1:** Significantly enriched bacterial taxa (from phylum to genus level) detected by LEfSe analysis.

Most abundant group	Level	Taxa	Relative abundance (%)		Sig.
			H	D	
D	Order	*Pseudonocardiales*	0.12	0.30	*∗*
		*Streptosporangiales*	0.01	0.08	*∗*
	Family	*Pseudonocardiaceae*	0.12	0.30	*∗*
		*Dermabacteraceae*	0.00	0.13	*∗*
		*Sporichthyaceae*	0.06	0.15	*∗*
		*Thermomonosporaceae*	0.00	0.04	*∗*
		*Nakamurellaceae*	0.01	0.04	*∗*
		*Promicromonosporaceae*	0.00	0.08	*∗*
	Genus	*Phyllobacterium*	0.00	0.27	*∗*
		*Dokdonella*	0.15	0.38	*∗*
		*Pseudonocardia*	0.08	0.21	*∗*
		*Brachybacterium*	0.00	0.13	*∗*
		*Collimonas*	0.06	0.19	*∗*
		*Promicromonospora*	0.00	0.08	*∗*
		*Actinomadura*	0.00	0.03	*∗*
		*Asticcacaulis*	0.02	0.04	*∗*
		*Nakamurella*	0.01	0.04	*∗*
		*Knoellia*	0.01	0.03	*∗*
		*Rhodomicrobium*	0.02	0.04	*∗*
H	Phylum	*Acidobacteria*	19.69	8.75	*∗*
		*Chlorobi*	0.17	0.05	*∗*
	Class	*Chlorobia*	0.17	0.05	*∗*
	Order	*Chlorobiales*	0.17	0.05	*∗*
	Genus	*Hartmannibacter*	0.05	0.00	*∗*
		*Variibacter*	1.78	0.73	*∗*

Significant differences are defined at *P* < 0.05 and an LDA score >2.0. Data show the average relative abundance of bacterial taxa in rhizosphere soils of *Panax ginseng* (*n* = 3). H: healthy plants; D: diseased plants; Sig.: significance; ^*∗*^*P* < 0.05.

**Table 2:** Significantly enriched fungal taxa (from phylum to genus level) detected by LEfSe analysis.

Most abundant group	Level	Taxa	Relative abundance (%)		Sig.
			H	D	
D	Order	*Capnodiales*	0.49	2.55	*∗*
	Family	*Pleosporaceae*	0.09	0.83	*∗*
	Genus	*Fusarium*	0.33	5.15	*∗*
		*Doratomyces*	0.38	3.84	*∗*
		*Alternaria*	0.09	0.83	*∗*
		*Acrophialophora*	0.01	0.41	*∗*
		*Cylindrocarpon*	0.04	0.26	*∗*
H	Phylum	*Basidiomycota*	11.54	4.46	*∗*
	Class	*Tremellomycetes*	7.14	1.05	*∗*
		*Pezizomycetes*	2.78	0.34	*∗*
	Order	*Filobasidiales*	4.20	0.50	*∗*
		*Pezizales*	2.78	0.34	*∗*
		*Trechisporales*	1.23	0.01	*∗*
		*Atheliales*	0.44	0.01	*∗*
		*Polyporales*	0.53	0.07	*∗*
	Family	*Polyporaceae*	0.52	0.07	*∗*
		*Atheliaceae*	0.44	0.01	*∗*
		*Piskurozymaceae*	3.89	0.46	*∗*
		*Piskurozymaceae*	3.89	0.46	*∗*
		*Hydnodontaceae*	1.23	0.01	*∗*
		*Hypocreaceae*	1.67	0.80	*∗*
		*Strophariaceae*	0.72	0.01	*∗*
		*Leotiaceae*	0.89	0.24	*∗*
		*Filobasidiaceae*	0.31	0.03	*∗*
	Genus	*Wilcoxina*	2.27	0.00	*∗*
		*Solicoccozyma*	3.89	0.46	*∗*
		*Trechispora*	1.23	0.01	*∗*
		*Phialocephala*	1.37	0.27	*∗*
		*Trichoderma*	1.56	0.64	*∗*
		*Hamigera*	0.50	0.00	*∗*
		*Ganoderma*	0.52	0.07	*∗*
		*Piloderma*	0.42	0.00	*∗*
		*Galerina*	0.39	0.00	*∗*
		*Neobulgaria*	0.32	0.02	*∗*
		*Scutellinia*	0.29	0.00	*∗*
		*Cryptococcus*	0.25	0.01	*∗*
		*Chalara*	0.23	0.02	*∗*
		*Arthrobotrys*	0.24	0.04	*∗*
		*Xenopolyscytalum*	0.21	0.00	*∗*

Significant differences are defined at *P* < 0.05 and an LDA score >3.0. Data show the average relative abundance of fungal taxa in rhizosphere soils of *Panax ginseng* (*n* = 3). H: healthy plants; D: diseased plants; Sig.: significance; ^*∗*^*P* < 0.05.

## Data Availability

The data used to support the findings of this study are included in the article.
